# Approach to procedural ventilation in a BPD infant undergoing hemodynamic assessment: a case report

**DOI:** 10.3389/fped.2026.1894996

**Published:** 2026-10-27

**Authors:** E. P. Filley, E. B. Mossad, M. K. Webb, N. M. Villafranco, R. Coleman, N. P. Varghese

**Affiliations:** 1 Texas A&M College of Medicine, Dallas, TX, United States; 2 Department of Pediatric Anesthesiology, Perioperative and Pain Management, Texas Children's Hospital, Baylor College of Medicine, Houston, TX, United States; 3 Division of Cardiology, Department of Pediatrics, McGovern Medical School, Houston, TX, United States; 4 Division of Pulmonology, Department of Pediatrics, Baylor College of Medicine and Texas Children's Hospital, Houston, TX, United States; 5 Divisions of Critical Care and Pulmonology, Department of Pediatrics, Baylor College of Medicine and Texas Children's Hospital, Houston, TX, United States

**Keywords:** atrial septal defect, bronchopulmonary dysplasia, cardiac catheterization, case report, pulmonary hypertension, ventilation

## Abstract

We present the case of a five-month-old premature infant with bronchopulmonary dysplasia complicated by atrial septal defect and pulmonary hypertension. Given the patient's age and comorbid chronic respiratory failure, cardiac catheterization was deferred and pulmonary hypertension diagnosis was made by echocardiography. Unfortunately, the child developed pulmonary edema and clinical worsening with initiation of sildenafil therapy. Initial measurements on right heart catheterization suggested more pulmonary vascular disease than sequela from a left-to-right shunt, which was inconsistent with clinical picture. Real time multidisciplinary collaboration prompted adjustment of ventilation to a bronchopulmonary dysplasia approach (higher tidal volumes, adequate positive end expiratory pressure) through the anesthesia circuit. This resulted in a more clinically consistent hemodynamic profile, suggesting need for atrial septal defect closure. This intervention allowed treatment of the child's pulmonary vascular disease without further pulmonary edema, thereby decreasing morbidity and mortality risks. This case report is a good demonstration of the measured and clinical effect of disease-specific perioperative ventilation adjustments on hemodynamics in the bronchopulmonary dysplasia population. It also highlights the need for multidisciplinary team approach for optimal outcome.

## Case presentation

We present a prematurely born infant with severe bronchopulmonary dysplasia (BPD) who remained in intensive care since birth. She had significant comorbidities which are common in this population, including atrial septal defect (ASD) and concern for pulmonary hypertension (PH). There was extensive multidisciplinary discussion regarding the best approach to treat PH in a BPD infant with a large (22 mm) secundum ASD with good rims. Due to technical concerns with ASD device placement in smaller infants and perioperative risks with anesthesia and procedures, cardiac catheterization was deferred to allow for sildenafil initiation. Unfortunately, initiation of sildenafil in BPD infants may be associated with significant risks including worsening hypoxemia from ventilation/perfusion mismatch, hypotension, and development of pulmonary edema. We present this case in which medical management resulted in worsening clinical findings, discuss ventilatory challenges imparted by heterogenous lung disease and their effect on accuracy of hemodynamic measurements in the catheterization lab.

The patient was a five-month-old female infant born at 29 weeks of gestation, with prenatal history notable for prolonged premature rupture of membranes, and postnatal complications including ileal atresia. The child was in critical condition in the neonatal intensive care unit due to severe BPD, chronic respiratory failure and pulmonary edema. Serial echocardiography suggested elevated right heart pressures and demonstrated a moderate left-to-right shunt across an ASD. However, given the child's young age and comorbidities of chronic respiratory failure and the risk of cardiac catheterization, hemodynamic assessment was deferred. PH was diagnosed by echocardiography and sildenafil (1 mg/kg every 6 h) was initiated. Unfortunately, the child clinically worsened, requiring increased respiratory support and initiation of diuretics. Chest imaging (Supplementary Figure 1) showed interval worsening of acute-on-chronic pulmonary edema, prompting concern for increased pulmonary congestion due to flow through the ASD. A chest CT ruled out pulmonary vein stenosis or abnormal venous drainage (1). Medical management of the pulmonary edema was attempted with fluid restriction and initiation of furosemide 2 mg/kg every 8 h, but imaging continued to show pulmonary edema and serial echocardiography continued to show left-to-right ASD shunt, right atrial and ventricular dilation, enlarged pulmonary arteries, and mild to moderate right ventricular dysfunction (Supplementary Figure 2). Tracheostomy was discussed, however there was significant concern that the respiratory symptoms were in part related to the pulmonary edema, exacerbated by ASD-related flow. Therefore, although she was considered young for ASD device closure, the inability to balance the pulmonary edema that resulted from concomitant sildenafil treatment and the observed right heart changes prompted discussion for intervention. She was referred for hemodynamic right heart catheterization with Heart Center-approved ASD closure criteria: pulmonary-to-systemic blood flow ratio (Qp:Qs) >1.5 and indexed pulmonary vascular resistance (PVRi) <6 WU*m^2^ (2).

At the time of catheterization, the infant was 51 weeks postmenstrual age and weighed 6.22 kg. She was on CPAP 10 cmH_2_O and FiO_2_ 0.3, on which capillary blood gas measured pH 7.46 and PCO_2_ 51 mmHg. Bedside assessment showed an irritable infant with moderately increased work of breathing. She remained fluid-restricted, on furosemide and sildenafil.

Following induction of anesthesia in the cath lab (using fentanyl 2 mcg/kg, Propofol 2 mg/kg and rocuronium 1 mg/kg), the patient was intubated and transitioned to an anesthesia ventilator (PIP 18, PEEP 5, FiO_2_ 0.5). Tidal volumes ranged 20–45 mL, which was 3–7 mL/kg. Initial hemodynamics showed Qp:Qs 1.3 and PVRi 7.5 WU*m^2^—findings inconsistent with the clinical picture of increased pulmonary blood flow. Real time discussion between the interventional cardiologist, anesthesiologist and pulmonary hypertension pulmonologist realized that the intraprocedural ventilation settings were likely inadequate for the child's physiology, and ventilation was then adjusted to reflect chronic BPD settings and her pre-procedural PEEP support (PIP 22, PEEP 10, FiO_2_ 0.85), and increase iNO to 40 ppm, which yielded tidal volumes of 10 mL/kg (3). On these settings, repeat catheterization showed Qp:Qs 1.5 and PVRi 4.1 WU*m^2^ (Table 1), meeting pre-defined criteria for ASD intervention. These changes are likely reflective of a lower PVR in response to increase FiO_2_ and iNO, as well as a better ventilator settings to accommodate the BPD impact. Closure of the defect was successfully performed using a 20 mm GORE® CARDIOFORM Septal Occluder via a modified 8-French Amplatzer™ sheath. The patient returned to the intensive care unit in stable condition. She was able to continue sildenafil treatment for PH without recurrence of pulmonary edema. Following the ASD device closure, the infant was gradually weaned from iNO and ventilatory support, and was maintained on oral sildenafil.

**Table 1:** Hemodynamics in two testing conditions.

	Variable (units)	Condition 1 (baseline #1; PEEP 5, iNO 20 ppm, FiO_2_ 50%)	Condition 2 (baseline #2: PEEP 10, iNO 40 ppm, FiO_2_ 85%)
Ventilator settings	PIP (cmH_2_O)	18	22
	PEEP (cmH_2_O)	5	10
	TV (mL)	45	60
	RR (bpm)	20	20
	Ti (sec)	0.8	1
	FiO_2_ (%)	0.5	0.85
	iNO (ppm)	20	40
	ETCO_2_	51	45
Arterial blood gas	pH	7.38	7.34
	PCO_2_ (mmHg)	54	52
	PO_2_ (mmHg)	190	344
	Lactate	1.3	1.2
	HCO_3_	29	27
	BE	2.9	1.7
Hemodynamic values	RV (mmHg)	68/10	54/12
	RFA (mmHg)	84/51/63	67/43/51
	PA (mmHg)	61/30/39	46/23/33
	PA wedge (mmHg)	8	8
	SVC saturation (%)	62	79
	PA saturation (%)	71	87
	RFA saturation (%)	97	99
	Qp:Qs	1.3	1.5
	PVRi (WU*m^2^)	7.5	4.1

PIP, peak inspiratory pressure in centimeters of water (cmH_2_O); PEEP, positive end-expiratory pressure in centimeters of water; TV, tidal volume in milliliters; RR, respiratory rate in breaths per minute; Ti, inspiratory time; FiO_2_, fractional inspired oxygen; iNO, inhaled nitric oxide in parts per million (ppm); ETCO_2_, end-tidal carbon dioxide in millimeters of mercury (mmHg); pH, potential of hydrogen; pCO_2_, partial pressure of carbon dioxide in mmHg; pO_2_, partial pressure of oxygen in mmHg; HCO_3_, bicarbonate; BE, base excess; RV, systolic/end-diastolic right ventricle pressure in mmHg; RFA, systolic/diastolic/mean right femoral artery pressure in mmHg; PA, systolic/diastolic/mean pulmonary artery pressure in mmHg; PA wedge, mean pulmonary artery wedge pressure in mmHg; SVC sat, oxygen saturation in superior vena cava; PA sat, oxygen saturation in pulmonary artery; RFA sat, oxygen saturation in right femoral artery; Qp:Qs, ratio of pulmonary blood flow to systemic blood flow; PVRi, pulmonary vascular resistance indexed for body surface area in Wood units × m^2^.

## Anesthetic and ventilatory considerations

Premature infants undergoing intervention require careful management of ventilation, thermoregulation, glucose balance, and blood pressure (4). Limited glycogen stores and altered cardiac compliance increase anesthetic risk, so glucose-containing fluids such as 10% dextrose-normal saline and blood pressure monitoring are essential.

In severe BPD, traditional ventilatory strategies may not be effective in the presence of heterogeneous parenchymal lung disease. A targeted approach focusing on higher tidal volumes (10–15 mL/kg), higher PEEP levels (often >10 cmH_2_O), longer inspiratory times, lower respiratory rates, permissive hypercarbia (PCO_2_ > 45 mmHg), and SpO_2_ goal 90%–94% may be more effective.

Volume-targeted ventilation may reduce lung injury and improve gas exchange. The primary goal is to maintain functional residual capacity (FRC) to reduce alveolar collapse and minimize air trapping (3).

## Discussion

BPD affects over 40% of infants born before 29 weeks and often leads to pulmonary vascular remodeling and PH (2). The interplay between lung disease and cardiovascular function complicates clinical management. PH in BPD is influenced by lung volume, oxygenation, carbon dioxide levels, and ventilation strategy (5). In our patient, the change in PVR and the candidacy for ASD device closure was accomplished by increase in FiO_2_ and iNO as well as changes in ventilator setting to reflect the BPD requirements.

This case illustrates how ventilatory settings during catheterization can distort hemodynamic assessment, through decreased lung recruitment and inadequate ventilation. Infants with BPD often live on the far right of the lung volume–PVR curve due to chronic air trapping. Standard anesthetic ventilator approaches of physiologic PEEP, normal tidal volumes and age-appropriate rates can exacerbate the variable obstruction, time constant heterogeneity and disparate inflation that characterize BPD. The resultant effect on the pulmonary vasculature is negative, stimulating vascular compression and hypoxemia- and acidosis-related constriction. Even if the blood gas is normal prior to taking measurements, over the course of the catheterization procedure, regional hypoxia and acidosis can result. Measurements may be inaccurate, underestimating Qp:Qs and overestimating PVR. Therefore, an individualized ventilatory approach that promotes alveolar recruitment and prevents regional acidosis is critical and should be favored over traditional infant ventilation strategies, which assume normal lung compliance and homogenous lung time constants (3).

Assuming the infant has achieved a chronic respiratory steady state, we recommend maintaining or approximating the BPD infant's pre-procedure chronic ventilator settings, with particular attention to adequate inspiratory/expiratory cycling time, PEEP and tidal volume. Multidisciplinary collaboration between neonatology, pulmonology, cardiology, PH, intensive care and anesthesia teams is crucial for optimizing respiratory status, improving procedural safety, and ensuring data integrity (5). Adjustment to BPD-appropriate ventilation led to data congruent with bedside findings and enabled successful ASD closure.

## Conclusion

In infants with BPD and PH undergoing catheterization, employing a ventilatory strategy that is specific to chronic BPD is imperative to ensure congruency between clinical assessment and hemodynamic measurement. Tailored ventilation strategies with higher PEEP, effective tidal volumes, and slower rates with adequate time cycling are essential to prevent air trapping and promote gas exchange. This will in turn minimize hemodynamic variables such as vascular compression and vasoconstriction, affording appreciation of the true hemodynamic state. This approach requires multidisciplinary discussion for optimal outcomes.

This case underscores the impact of ventilation on hemodynamics and the need for physiology-specific ventilatory support. The ventilatory changes to reflect BPD requirements resulted in a better clinical picture that confirmed candidacy for ASD device closure. These changes are not universal to all infants with BPD and should be approached with individualized care in a multidisciplinary approach.

## Data Availability

The datasets presented in this article are not readily available because no identifiable data included. Requests to access the datasets should be directed to mossad@bcm.edu.
