# Supplementary material for: Approach to procedural ventilation in a BPD infant undergoing hemodynamic assessment: a case report
Source: Front Pediatr. 2026 Oct 27;14:1894996. doi: 10.3389/fped.2026.1894996 (PMC13749484; doi:10.3389/fped.2026.1894996)
Supplement: Supplementary file 1 [file Supplementaryfile1.docx]

**Supplemental Figure 1: Chest imaging.**

2A: Anterior chest Xray consistent with BPD.  There is heterogenous opacification with areas of hyperinflation and atelectasis and overlying diffuse haziness consistent with diffuse pulmonary edema.

2B: Computed chest tomography of the lungs showing severe heterogeneity of lungs with posterior lung atelectasis, regional pulmonary edema and prominent vasculature. Pulmonary veins are unobstructed and draining normally.

C
