# Supplementary material for: Approach to procedural ventilation in a BPD infant undergoing hemodynamic assessment: a case report
Source: Front Pediatr. 2026 Oct 27;14:1894996. doi: 10.3389/fped.2026.1894996 (PMC13749484; doi:10.3389/fped.2026.1894996)
Supplement: Supplementary file 2 [file Supplementaryfile2.docx]

**SUPPLEMENTAL** **Figure 2: Serial echocardiograms throughout hospitalization.**

Echocardiogram clips show left to right atrial level shunt and dilated and hypertrophied right ventricle.  *ASD* atrial septal defect, *RV* right ventricle, *LV* left ventricle.
